# Inaction, under-reaction action and incapacity: communication breakdown in Italy’s vaccination governance

**DOI:** 10.1007/s11077-021-09427-1

**Published:** 2021-06-15

**Authors:** Katie Attwell, Tauel Harper, Marco Rizzi, Jeannette Taylor, Virginia Casigliani, Filippo Quattrone, PierLuigi Lopalco

**Affiliations:** 1 School of Social Sciences, University of Western Australia, 35 Stirling Highway, Crawley, WA 6009 Australia; 2 Wesfarmers Centre of Vaccines and Infectious Diseases, Telethon Kids Institute, Northern Entrance, Perth Children’s Hospital, 15 Hospital Avenue, Nedlands, WA 6009 Australia; 3 Law School, University of Western Australia, 35 Stirling Highway, Crawley, WA 6009 Australia; 4 Department of Translational Research and New Technologies in Medicine and Surgery, University of Pisa, Pisa, Italy

**Keywords:** Vaccination policy, Mandatory vaccination, Policy governance, Government inaction

## Abstract

This article explores why governments do not respond to public compliance problems in a timely manner with appropriate instruments, and the consequences of their failure to do so. Utilising a case study of Italian vaccination policy, the article considers counterfactuals and the challenges of governing health policy in an age of disinformation. It counterposes two methods of governing vaccination compliance: *discipline*, which uses public institutions to inculcate the population with favourable attitudes and practices, and *modulation*, which uses access to public institutions as a form of control. The Italian government ineffectively employed discipline for a number of years. Epistemological and organisational constraints stymied its efforts to tackle a significant childhood vaccination compliance problem. With a loss of control over the information environment, vaccinations were not served well by exogenous crises, the sensationalism of the news cycle and online misinformation. Hampered by austerity, lack of capacity and epistemic shortcomings, the Italian government did not protect the public legitimacy of the vaccination programme. Instead of employing communications to reassure a hesitant population, they focused on systemic and delivery issues, until it was too late to do anything except make vaccinations mandatory (using modulation). The apparent short-term success of this measure in generating population compliance does not foreclose the need for ongoing governance of vaccine confidence through effective discipline. This is evident for the COVID-19 vaccination campaign, with many Italians still indicating that they would not accept a vaccine despite the devastation that the disease has wrought throughout their country.

## Introduction

Childhood vaccination has saved countless lives, prevented large-scale human suffering, reduced public health expenditures and preserved economic and social functioning. Its mass effect, known as ‘herd immunity’ or ‘community protection’, protects vulnerable individuals when coverage rates reach around 95%, the ‘aggregate target compliance’ (Weaver, 2014), which represents a key public health goal. Knowing that some of the population cannot be vaccinated due to age or health, and others will be poorly reached by vaccination programmes, governments have a strong interest in promoting acceptance and limiting refusal. Maintaining confidence in vaccinations is a crucial mechanism for avoiding more coercive policy instruments.

Since 2015, several high-income jurisdictions have made their childhood vaccination programmes more coercive. These include Australia, France, California and Italy (Attwell et al., 2018), and more recently Germany (Eddy, 2019) and additional US states (New York State, 2019, Maine 2019). None of these jurisdictions invested in any large-scale public communications to persuade the population of the benefits of vaccinating before making their policies more coercive. And all these developments occurred ahead of a vaccination programme for COVID-19, which will require high compliance. Accordingly, the time is ripe for questioning why governments do not adequately address vaccine confidence through non-mandatory measures while they still can.

The Italian experience provides a particularly salient case for considering counterfactuals and the challenges of governing health policy in an age of disinformation. Italy faced a vaccination compliance problem for half a decade, with plummeting coverage rates and several indicators of low population confidence. This paper asks, using the Italian case study: Why don’t governments respond to public compliance problems in a timely manner with appropriate instruments, and what are the consequences of their failure to do so?

## Literature review

To address our research question, we build upon four distinct bodies of literature. The first is the interdisciplinary vaccination social science literature, which considers factors contributing to under-vaccination and strategies to address it. The other three literatures come from the policy sciences. Literature on compliance contextualises vaccination as a behaviour that governments must extract from populations in order to maintain social and economic function, yet extant works rarely focus on governments’ choices and actions. Literature on policy instruments and governance tools orients our attention to the mechanisms by which governments can extract compliance, yet these have been under-theorised in the context of vaccination. Finally, literatures on inaction and under-reaction shed light on why governments might not address non-vaccination with appropriate policy instruments before compliance becomes such a problem that coercive instruments are mobilised.

### Vaccination social science

The interdisciplinary literature on under-vaccination rarely conceptualises it as a compliance problem (for one exception, see Gofen & Needham, 2015). However, the necessity of high coverage underpins almost all research, and scholars’ exhortations that health professionals, public health actors, community groups and governments promote vaccine acceptance (Opel et al., 2013; Leask et al., 2012; Saeterdal et al., 2014). Less coercive policy instruments are generally preferred to coercive policies (Leask & Danchin, 2017; Navin & Largent, 2017; Omer et al., 2019), despite many jurisdictions at least partially coercing populations to vaccinate (McCoy, 2019; Gravagna et al., 2020). More recent work on mandates is starting to consider vaccination an exemplar of a compliance problem centred on vaccine refusing families (Attwell & Navin, 2019; MacDonald et al., 2018). This opens up questions regarding governments’ failures to utilise less coercive policy instruments in the periods prior.

### Compliance with public policies

Public policy literatures on compliance have predominantly focused on the practices, experiences, state of mind and capacities of the governed (Meier & Morgan, 1982; Weaver, 2014; Schneider & Ingram, 1990; Levi, 2009; Nielsen & Parker, 2009). Scholars have paid less attention to the role and response of governments, and such attention has often focused on enforcement agents and frontline personnel (Edwards, 2006; Gofen & Needham, 2015; Hasenfeld & Weaver, 1996), rather than those managing the systems tasked with extracting compliance. Building on Gofen and Needham’s (2015) recognition that governments’ roles need more attention, this article considers the lack of appropriate action by government in addressing a large-scale compliance problem.

### Policy instruments, strategies of governance and coercion

Scholars of governance have paid attention to the strategies that governments can employ to extract compliance with vaccination programmes. McCoy’s (2019) study of Australia, the USA and the UK employed Vedung’s (1998 [2011]) tripartite framework of carrots, sticks and sermons. Attwell and Smith’s (2018) theoretical commentary utilised the five-pronged approach advanced by Mols et al. (2015), consisting of hierarchy, markets, networks, persuasion and nudge. Relative coerciveness is a key focus when distinguishing between compliance mechanisms available, and the desirability of one mechanism over another. Tackling vaccine refusal often involves *persuading* individuals with targeted communications (Attwell & Freeman, 2015; Nowak et al., 2015), or *mandating* their behaviour by imposing unavoidable consequences for refusal (Attwell & Navin, 2019; MacDonald et al., 2018).

That these strategies are not regarded as equal is evident in the robust debates regarding mandatory vaccination. Common critiques reveal many reasons to prefer persuasive communication campaigns: vaccine mandates can punish children for their parents’ decisions and exacerbate inequalities (Leask & Danchin, 2017), backfire and generate psychological ‘reactance’ amongst target populations (Betsch & Böhm, 2015) and fail to change the entrenched behaviours of committed refusers (Helps et al., 2018). By contrast, well-researched and targeted public communications can change attitudes and beliefs (Attwell & Freeman, 2015) and build and protect public trust. High-functioning public health systems can successfully maintain voluntary compliance, with the UK as an exemplar (Elliman & Bedford, 2013; McCoy, 2019).

To illuminate the choices facing public officials in Italy and beyond, we employ Deleuze’s concepts of discipline and modulation as approaches to governance. These help us to make deeper sense of vaccine voluntarism centred on effective public engagement on the one hand and vaccine mandates on the other. Discipline and modulation allow us to treat the continuum of coercion (Attwell et al., 2018; McCoy, 2019) like an iceberg, exploring the submerged properties of vaccination regimes.

Three decades ago, Deleuze (1992) identified that ‘societies of control’ are in the process of replacing ‘disciplinary’ societies. Disciplinary societies, as described by Foucault (1975), focus on developing individuals who are ‘disciplined’ (by enclosures such as schools, prisons and factories) to behave obediently. Viewing vaccination through this lens, voluntary regimes can thrive when governments maintain successful discipline through public communications and effective spheres of influence. For example, vaccination enjoys widespread acceptance in the UK because of its ubiquitous reinforcement through individuals’ encounters with public health systems (McCoy, 2019). Hence, a successful ‘disciplinary’ approach convinces individuals that vaccinations are necessary, using education, public institutions and public communication tools to ‘normalise’ compliance. Such norms are inherently powerful (Winter & May, 2001).

‘Control societies,’ on the other hand, maintain orderly function through ‘modulation’—changing the conditions around the individual—to compel compliance. Disciplinary societies are regulated by ‘watchwords’ that stand in for social conscience, whereas modulatory societies use ‘passwords’ to control through access (Deleuze, 1992, p. 5). Vaccine mandates deny unvaccinated children access to shared public spaces such as schools and kindergartens. Vaccination is a password for entry, and regimes do not (on the face of it) need the disciplinary resources of the state to shape population behaviour when control over access to public institutions performs this function.^1^

It appears that in some jurisdictions, failing discipline has necessitated the resort to mandates. However, it also appears that disciplinary instruments have not been exhausted as the vaccination compliance problem unfolded in many jurisdictions. This brings us to the final literature that informs our study, which is the literature on inaction and under-reaction.

### Inaction and under-reaction

We are interested in why governments fail to intervene to maintain ‘discipline’ in voluntary vaccination systems, such that they eventually make vaccinations mandatory. McConnell and t’Hart (2019, 648) define inaction as an “instance and or pattern of non-intervention” in relation to an issue that falls within the policymaker’s jurisdiction. Researchers have previously identified three key reasons that governments fail to act: exclusionary agenda setting (Cobb & Ross, 1997); bias (Wolfinger, 1971; Bachrach & Baratz, 1963); and the perception that wicked problems are too difficult to solve (Levin et al., 2012). McConnell and t’Hart (2019) delineate *ideological* inaction (characterised by attitudes about state involvement and role), *imposed* inaction (institutional stalemates and architectures), *reluctant* inaction (lacking tools, resources and options) and *inadvertent* inaction (stressed policymakers cannot see what’s important).

Maor’s (2014) scholarship on policy under-reaction is also useful. Maor describes under-reaction as a ‘systematically slow and/or insufficient response … to increased risk or opportunity’ (p. 426). Understanding whether policymakers accurately perceive risk and whether they face internal or external constraints to action can shed light on how public officials perceive vaccine confidence crises and the availability of public communication resources. Maor also notes that future research should engage with how cognitive, emotional, organisational and institutional factors interact (p. 440), which we explore in this study.

## Methods

### Italian case selection

Our research question asks why governments fail to adequately mobilise non-coercive instruments for vaccination policy compliance in a timely fashion. The sheer number of jurisdictions who have recently made vaccination more mandatory without prior public communication campaigns suggests a wide array of choices for case studies, hence the appeal of comparative work. However, we dive into a single case that we argue can be instructive for others. Single case studies enable researchers to analyse the broader social, economic and governance context and conduct process tracing (George & Bennett, 2005). Italy functions as an emblematic *instrumental* case study for answering our research question; equally, Italy is an *intrinsic* case of interest in its own right (Baxter & Jack, 2008), particularly because multiple modes of governing vaccination have been attempted during its recent history.

### Case analysis

We utilised a synthetic research design, drawing upon primary sources and Italian and international scholarship. Legal records and policy documents were examined. Following ethics approval, key informant interviews were conducted with experts prominently involved in, or privy to, decisions taken around addressing Italian vaccine confidence. These included two Ministry of Health (MH) employees, two technical experts (TE) and two academics providing vaccination expert advice and participating in civil society advocacy (AC). Interview transcripts were analysed in NVivo to arrive at a framework for explaining the lack of appropriate action, employing discipline and modulation as modes for governing vaccination compliance.

## Case study

### Building a robust voluntary vaccination system?

Our data indicate that Italian public health officials were consistently mindful of the need to maintain high vaccine coverage, but had prioritised *voluntarism* and *enhanced service delivery* over governing public confidence. This manifested as unthinking or aspirational discipline, in which public officials imagined that a mature population would continue to choose vaccination. Accordingly, they did not augment failing mandates with other strategies to maintain social norms.

Italy’s 2017 mandates, though headline grabbing, were not entirely new. Authorities previously required parents to vaccinate their children against diphtheria, polio, tetanus and hepatitis B in order to enrol in school. These legacy mandates were ostensibly an incentive/sanction tool (Schneider & Ingram, 1990), yet cued government provision of vaccinations, rather than community uptake (Moran et al., 2008). They made access to school a point of control but did not employ it effectively and thus can be understood as a weak form of modulation. This is evident in a long history of non-enforcement, culminating in the Supreme Court ruling in 1999 that Italy’s constitution prohibited excluding children from education (Crenna et al., 2018). Italy moved further to quasi-voluntary vaccination. Officials rarely applied the fines that remained available to sanction those not accepting ‘mandatory’ vaccines (Attwell et al., 2018), perceiving them as low cost opportunities for refusers to ‘purchase’ non-compliance (TE1). Thus, Italy’s vaccine mandates were reduced in practice to nothing more than an ‘authority tool’ (Schneider & Ingram, 1990): government says you must, so it is expected that you will. This was not going to be sufficient.

When it came to Italy’s recommended vaccines, there was not even the force of an authority tool, and no targets or uptake policies until 1995, when the Ministry of Health established that Italy’s regions should begin offering and promoting free MMR and pertussis vaccines. The 1998–2000 National Health Plan aimed to reach 95% coverage, and the National Vaccination Plan of 1999 sought to apply unified strategies of delivery and promotion, with the aim of eventually removing the mandate altogether (Ministry of Health, 1999). Treating all vaccines as important did improve uptake of ‘voluntary’ vaccines. So did the introduction of hexavalent vaccines, which combined some voluntary antigens with mandatory ones (Bonanni & Bergamini, 2001). Having successfully increased coverage through supply-side tweaks, public health officials began to consider officially withdrawing mandatory vaccination (Bonanni et al., 2015). The regional government in Veneto took the lead in 2007, committing to communicating the benefits of vaccines to the population (Russo et al., 2009). But vaccine voluntarism was about to become sorely tested.

### An unfavourable economic and sociopolitical context

Like many other countries in Europe, Italy experienced a turbulent period following the 2008 Global Financial Crisis. In 2011, a new government was appointed, composed of non-partisan, largely non-elected and highly qualified professional figures, earning it the title of ‘technocratic’. Its principal goal was to bring the country back to compliance with stringent EU parameters on public budget deficit. These politics of budget tightening, which became simply known as ‘austerity’, were particularly harsh on southern countries including Italy (Perez & Matsaganis, 2018). When the newly elected centre-left Italian government took power in April 2013, austerity had become an undisputable mantra, with no political or legal alternative (Farrand & Rizzi, 2019).

Austerity severely impacted Italy’s public sector, with public health cuts of up to 2.5 billion euros between 2012 and 2014 (Ferrè & Ricciardi, 2015; OECD et al., 2017). Technocratic rule—combined with reduced spaces for democratic engagement and austerity measures—fuelled a strong anti-expert sentiment throughout Europe (Weimer & de Ruijter, 2017). In Italy, this sentiment, together with an increasing precariousness of economic welfare, contributed to the dramatic rise of the anti-establishment Five Star Movement. In this precarious political scenario, immunisation compliance suffered from the public’s loss of trust in institutions and expertise. Trust is central to compliance with public policies in general (May, 2004; Lawson, 2007), as well as for vaccination (Casiday et al., 2006; Evans et al., 2001; Peretti-Watel et al., 2019).

### A crisis in confidence

A series of vaccine-related crises further undermined population compliance. In 2014, deaths among elderly Italians were incorrectly linked to the *Fluad* influenza vaccine. Extensive media coverage publicised the crisis, but not the investigations that cleared the vaccine (Signorelli et al., 2015; Odone et al., 2015). A series of court cases also cast vaccination in a negative light. Tribunals in Rimini and Milan awarded payouts to parents who claimed that vaccines had caused their children’s autism. While both decisions were overturned on appeal (Sentenza n. 148/2010, 2012; Sentenza n. 2664/2014, 2014; Ministry of Health, 2015), authorities could not put the genie back in the bottle, and Italian-language internet searches about MMR and autism skyrocketed (Aquino et al., 2017). A public prosecutor in Trani ordered an inquiry into the link between vaccines and autism, which ultimately ruled out any correlation (Rizzo & Rota, 2016), but was yet another bad news story. Pharmaceutical companies were also rendered suspect because Italian media emphasised negative findings from an anti-trust report about the resourcing of Italy’s state-funded vaccines (AGCM, 2016).

The Italian population’s vaccine confidence could not withstand this battering. During 2010–2015, compliance with the schedule decreased significantly. MMR vaccination coverage decreased by 5.4%, hitting a low of 85.2%. Notably, the largest decrease (− 1.7%) was registered in 2013 and the following two years, aligning with the Rimini court case and the *Fluad* scare.

For those in the health bureaucracy tasked with maintaining compliance, Italy’s communication environment posed additional challenges. Anti-vaccination sentiment had infiltrated mainstream and social media by 2012 (Aquino et al., 2017) and was exacerbated by false balance, whereby the media gave equal airtime to vaccine denialists and experts (Bonanni, 2018). In such a context, ‘aspirational’ discipline and expected population conformity were not strong enough to maintain vaccination rates. However, revision of these methods would prove exceptionally difficult.

### Overview of responsibility, action and preparedness

The Italian government was facing a compliance crisis which, like many, was perhaps only observable in retrospect. Nevertheless, during 2012–2017, Italian authorities did not implement a communication campaign to address vaccine confidence or the vast swathes of vaccine misinformation circulating online. For the global community of practice working on vaccine uptake, a communication strategy is imperative in these conditions (MacDonald et al., 2018); from the perspective of implementing ‘discipline’, it is also highly important. Schneider and Ingram (1990) note that ‘authority tools’ need buttressing by interventions to increase the capacity of populations (including providing information about why certain behaviours are necessary) or through symbolic and hortatory tools that connect desired behaviours to targets’ values. Italy had none of this.

Given Italy’s decentralised health system, we sought to confirm whether the national Ministry of Health was indeed responsible for maintaining public confidence in vaccination. Since 2001, Italy has increased regional autonomy in health policy through changes to the national constitution (Title V, Part Two). The Ministry retained responsibility for setting the national agenda and steering, while regional governments fund, coordinate and deliver programmes (Curto et al., 2014). The Ministry is divided into a number of divisions, with vaccination sitting in the Prevention portfolio. Informants explained that communications fall under the regional remit, and one linked this to a limiting diffusion of responsibility (TE2). However, all regarded a national campaign as a significant priority:

A national campaign is even more important. Because we are one country. And so to have one vision, to build to one belief [amongst the populace], it is important that we have a common and harmonised tool (MH2).

All informants saw the national Ministry of Health as responsible for maintaining public confidence, although one added that support from other agencies was essential, and “that *all* the bigger institutions should” speak with a single voice, “like a chorus” (TE2). This demonstrates how effective discipline should work—states relying on social norms need to use the entire apparatus to reinforce them, with buy-in from as many groups as possible. However, this was going to be a challenge even within the Ministry. The informant continued: “I’m not sure that at the time the Ministry of Health was really ready for a communication campaign… I consider the Ministry of Health at that time [to be] very bureaucratic.”

### Multidimensional drivers of inaction

The remark that the Ministry was not ready for a communication campaign might imply that government actors were merely unprepared for execution. However, our multidimensional analysis illuminated two sets of factors that went far beyond preparedness. *Epistemological constraints* pertained to the knowledge of key parties. *Organisational constraints* pertained to the business of governing.

#### Epistemological constraints

Italian authorities tasked with maintaining vaccination compliance did not understand the drivers of vaccine refusal, nor the best strategies to address it. This was compounded by a dearth of appropriate research into how the population acquired *their* knowledge. “The fake news was very important for the vaccine hesitancy,” one informant told us. “Because the medical doctors and the public health people didn’t realise that the way the people … get information was very different, was changing” (AC2).

Lack of knowledge made it difficult to replace ‘aspirational but unthinking’ discipline with a more targeted approach. Not understanding their audience, the Ministry did not know how to speak to them, nor *where* to speak to them. In the aftermath of the Rimini court case, Ministry staff fronted traditional media and set up a telephone hotline, describing ‘comprehensive and empathic’ responses to phone calls and written communications (MH2). However, they failed to campaign on digital and social media, lacking the ability and an understanding of “the significance of different kinds of means or tools can have on the Italian population, depending on our culture.” (MH2).

These epistemological limitations comprised only half the knowledge problem stymieing a more concerted disciplinary approach. Beleaguered officials also believed their audience was uninterested in the knowledge they sought to convey. Ministry staff described a history of issuing ‘institutional communications’ trusted by the public. Now, such communications were met by ‘aggressive’ anti-establishment feeling and scepticism towards science (MH1). Ministry staff’s perceptions about their audience’s knowledge were also a constraint to their action—as we shall revisit later.

#### Organisational constraints

Despite Ministry staff’s desire to address vaccine confidence with a public communication campaign, they lacked capacity. Most crucially, they lacked manpower, which came down to money. “The investment in communication of the Ministry of Health, especially in Prevention, is extremely poor,” one informant told us.

There are no resources, there are not even so many people involved in the Prevention Office … They have to deal with an enormous amount of practises, information, meetings. So I would say in general that the Prevention sector in the Health Ministry is heavily underpowered.

This lack of staffing was the responsibility of the elected government (AC1).

The Ministry also lacked, in the local and literal translation, “a wallet.” “So they have to receive funds from the Ministry of Finance.” (TE2). A large multimedia vaccination communication campaign would have been well beyond the fiscal capacity of the Prevention portfolio or the Ministry, requiring external funds. Ministry staff told us, rather jadedly, of their repeated attempts to acquire them.

Each year, all the Ministries had to propose to our government… a list of issues for what they would like a public communications campaign, [which] will be funded the year after. And I know that each year we made a request for [a] communication campaign on vaccinations. And this year… we will do the same. But I don’t know if it will be considered a priority (MH2).

There were additional means by which “the bureaucracy propose… to the political level … some choices to do through the year,” the informant continued.

It usually happens in a general way because we, as officials, we realise that there is an issue. We always wrote officially to the Minister, to the political level: ‘This is the issue; this is what we propose.’ And often we also say, ‘This is the money that is necessary to solve this problem.’ There are also other opportunities during the year. At the end of each year, the government release the budget decree, and so they ask us what are our proposals for this year… But only a part of these proposals are funded (MH1).

However, informants from outside the Ministry offered more nuanced perspectives. One suggested that a communications campaign took time rather than money; baseline Ministry staff could have managed it in the same way that they spruiked the importance of vaccines in the public sphere (AC2). Another suggested that having communications experts on staff would generate Ministry capacity.

Our data indicated a lack of leadership and accountability when it came to addressing vaccine confidence. This is not to say that those with power did not care, but rather that they were preoccupied. Towards the end of the study period, Italy’s vaccination schedule was being revamped to include more vaccines and more government funding (Bonanni et al., 2017). These significant changes were occupying both government and civil society actors who might otherwise have pursued funding for the vaccine confidence problem more vociferously (AC2). This was a continuation of the previous decade’s focus on systemic factors. Ministry staff pointed out that only in 2016 did a new law pass for robust funding of vaccines. “[It] contains a quota of money just for vaccinations. But not for vaccination campaigns” (MH2).

While several informants spoke glowingly of Dr Guerra, former leader of the Department of Prevention, for being among the key policy entrepreneurs to reorient Italy’s vaccine policy to mandates, neither Dr Guerra nor the (then) Health Minister, Beatrice Lorenzin—similarly lauded as a champion—mobilised a campaign to address vaccine confidence. By the time the new schedule had been adopted—which may have been a logical, if late, moment—an impending national election dissuaded political impetus and discouraged those who might otherwise have sought action. “Nobody was able to take this decision,” one informant reflected. “When there is a severe problem … often at the Ministry of Health or at the greater level, it’s difficult to find somebody that takes the responsibility to answer, and use a lot of money, for instance, to answer … a problem like this” (TE2).

## A communication problem

The absence of funding to manage discipline for vaccination needs to be understood in the context of austerity. But austerity and technocracy also contributed to public distrust, likely rendering it difficult for government actors to claim legitimacy even with abundant resources. Interviewees situated these constraints in an interpretative context that helped them to *explain* what had (not) happened and *justify* the eventual resort to mandates. They did so by two interconnected narratives: *Italians are bad at communications*, and *communications don’t work anyway*.

The sentiment that Italians are bad at communications was reflected widely. As a culture, Italians didn’t see communications expertise as a specialist skill. This was not just a government problem. “I have difficulties to … identify key communication persons in *all* the main Italian institutions,” one informant commented.

…I think that only [in] the private sector you can see wonderful campaigns. So, one who is selling vaccines, obviously, produce[s] wonderful campaigns. They invest money and resources. [In the public sector] for technical level for vaccination, yes. For communication? We still have to find… You have to keep a lot of budget, also, [directed] to them (TE2).

If public communication was an Italy-wide problem, then the Ministry of Health epitomised it, with the informant also noting that “at the regional level you can see something better.” The Ministry had been involved in two health communication campaigns widely regarded as fiascos, which informants recounted to illustrate this deficit. One campaign exhorting women to start breeding, lest their biological clocks run out, was roundly criticised for its patriarchal tone (Pianigiani, 2016). Another used two families to illustrate how to live a healthy lifestyle; the healthy family was white, while the family making poor lifestyle decisions was not (Rubino, 2016). “So you can imagine—racism” (TE2).

Even beyond such problems, institutional communications were not reaching audiences through the most appropriate channels.

In the past the public administration, in communication, was a bit slower than the new media… Older people watch television, people of my generation… The young generation use websites and the web as a source, but the new generations only uses social media… The Ministry of Health, [and] in general the public sector in Italy, is reacting now [in that space]. But three, four, five years ago, the public sector was slower than the new media (MH1).

Even at the time of the interview (November 2018), the Ministry’s website had only recently been updated for mobile phone navigation.

When it came to communication strategies to promote vaccination compliance, informants drew comparisons to the UK, the case par excellence for successful discipline.

[V]accination programs are managed, from the communication point of view, by an office that has experts in communication, and they build the communication campaigns there … so they are really well organised. We have always thought in Italy that communication is something you can do in [your] free time, or without a very structured office where there are experts in communication that know how to speak to the population. So I see a dramatic lack of organization of communication (AC1).

This informant depicted a “cultural problem” that included lack of communications training in medical schools and lack of a “strong position” in public health communications traditionally. “We have to learn from other countries like U.K.,” he concluded.

However, discussions of cultural problems with communication often morphed into problems with the Italian communication *audience*. A Ministry informant also reflected, unprompted, on the UK, but with less conviction that Italy could import British practices.

I know, for example, that U.K., when there is any incident or episode regarding vaccination strategy that could impact on vaccination strategies and policies, they immediately put [out] a press release. But I am not sure that it is enough (MH2).

The informant was alluding to the idea that the Italian population might not comply because of its *own* communications problem. This informant saw Italian culture driving misinformation, anti-establishment rhetoric and distrust in scientific expertise. While the best vaccination social science urges that interventions to build compliance be locally researched and targeted (Butler and Habersaat, 2019), Ministry staff constructed and essentialised an audience incapable of listening to them. They concluded that a campaign may have been insufficient to address the falling rates of vaccination. This sentiment likely drew on the case of Veneto, which had abolished vaccine mandates in 2007. “They invested a lot of money [in communications], and the coverage rates [still] went down more than the other regions.” (AC2).

On the inevitability of the new mandates, informants were in broad agreement.

So, it seems that such communication was not done enough. I don't have the counter proof that if they would have done good communication, we would have raised the coverage without the mandatory law. … [V]accine hesitancy is a general problem everywhere. So I'm not sure that that that a good communication could... a good communication campaign, even if done like in the U.K., would have reversed completely the situation (AC1).

Yet in discussing the new vaccine mandates, Ministry officials emphasised their communicative power.

… [M]andatory vaccinations can appear for other cultures and countries something very strange. [But] … I think that in Italy it is a good strategy. Because Italians need that the institution take the responsibility and tell them what they have to do … There is a part [group] of people that need someone else, the institution, who has the authority, saying to them what they have to do (MH2).

The Italian government saw that mandates could speak to Italians—tell them ‘what they have to do’—in a way that would finally bring about compliance. Other opportunities to ‘speak’ were not attained and perhaps would not have worked anyway, due to (perceived) deficits in the population. Mandates, however, could correct the problem. And so discipline gave way to the access control of modulation.

## Italy’s childhood vaccine mandates: redux

In July 2017, Italy’s government passed a law extending mandatory vaccinations from four to ten antigens (Epicentro, 2017) with vaccinations required for childcare and kindergarten (D’Ancona et al., 2018). Parents of older unvaccinated children are subject to fines of 100-500 euro (Crenna et al., 2018). In a belated nod to discipline, the law explicitly made the Ministry of Health responsible for spreading the culture of vaccination amongst the population; it later ran a multimedia campaign featuring a sportsman and an astronaut and introduced vaccine-promoting content to its website. The government allocated an additional €200,000 to a collaboration with the Ministry of Education to develop initiatives for schoolteachers and students to promote vaccination (Casula & Toth, 2018).

In the 6 months following the new law, vaccination rates increased for both Polio (+1.2%), used as a proxy for the hexavalent vaccine, and MMR (+4.4%), as compared to 2016 (D’Ancona et al., 2018). Coverage rates continued to climb in 2018, even for non-mandatory vaccinations, which authorities claimed as a win for vaccine confidence (D’Ancona et al., 2018). Italy’s public health officials, technical experts and professional societies regard the new mandates as a resounding success. The spectre of guilt hovers over some conclusions, however. A team of public health scholars reflected in *Lancet Infectious Diseases* that while their Australian counterparts were critical of that country’s mandatory vaccination laws, the Italian scientific community broadly supported theirs. “Is this a sign of resignation?” they asked. “Or worse, is this an admission of guilt for having failed to promote the individual and social value of immunisation through health education, empowerment, and people-centred prevention?” (Signorelli et al., 2018).

## Discussion

We have sought to explain why public officials did not respond in a timely manner, with appropriate policy instruments, to a crisis of vaccination compliance. Although we could have asked this question of a number of jurisdictions, we focused on Italy, with its clear confidence crisis, years of in(adequate) action to maintain discipline and its ultimate recourse to modulation, albeit with a belated investment in discipline.

Our key finding pertained to a fatal dearth of *disciplinary capacity*. There was institutional blinkeredness—a sense that vaccination would simply continue without the functionality of mandates. But there was also an intrinsic lack of capacity to reorient to a more effective form of discipline. Officials blamed the audience for not listening to them, while neoliberalism, austerity and social media made it distinctly difficult for officials to conduct discipline effectively.

Limitations within the Ministry, and the significance of these to our findings, indicate the contribution of (in)capacity to insufficient action. Policy capacity is a critical determinant of government performance (Bakvis, 2000; Meckling & Nahm, 2018), and Howlett and Ramesh (2016) identify three competences that define it: (1) *analytical* competences that enable policy options to be effectively generated; (2) *managerial* competences that allow resources to be effectively used for addressing policy issues; and (3) *political* competences that equip policymakers and managers with the support and autonomy to develop and implement ideas and programmes. Our findings map onto several of these. Managerial competences connect to the Ministry’s administrative resource capacity (e.g., insufficient staffing levels). Political competences include organisational political capacity: low levels of support from the political class likely explain why the Ministry failed to receive sufficient funding. Resourcing problems may also lie with the limited “political-economic capacity” of the national sector at a systems level in a decentralised system. While the importance of communication is supported by all three types of Howlett and Ramesh’s competences, it is particularly relevant under managerial and analytical competences. The latter includes the communication skills of individual government actors, the information and e-services architecture of their organisations, and the institutions and opportunities for knowledge mobilisation in a society, such as the lack of communication training in medical training (called knowledge system capacity). These competencies were all insufficient to provide government with the capacity to maintain discipline regarding vaccine confidence.

Viewing our findings through the prism of inaction, they map onto several extant categories. McConnell and t’Hart (2019) situate *ideological* inaction within divergent perspectives on the role of governments in solving social problems. However, ideology also characterises Italy’s prevailing voluntarist spirit regarding vaccination, which was replaced by a paternalistic (or defeatist) ideology that Italians need telling what to do. *Imposed* inaction is evident in the lack of funds available from executive government, which could also be characterised as *reluctant* inaction during a period of austerity. Finally, *inadvertent* inaction emerges through officials’ preoccupation with the new vaccine schedule, and supply-side tweaks.

Mapping our findings on to Maor’s (2014) under-reaction schema is equally demonstrative of multi-dimensionality. Did public officials understand the risks they faced? Experts in the Ministry did. Were constraints to appropriate action internal or external? Here, it becomes more difficult. Habits and patterns within the Ministry clearly contributed, but relations between the Ministry, other departments and elected government blocked access to vital resources. Maor notes that mental constructs and cultural traits can play a key role; the construction of the Italian audience as deficient is salient here, while EU-mandated austerity and declining public trust added weight to such perceptions. An area for further research is the persistence of ‘broken instruments’ in allowing officials to think that compliance was in hand. Italian officials had governed vaccination through mandates for decades, and did not remove or replace them. It is likely that these broken instruments lulled authorities into a false sense of security, and hence an insufficient grasp of what discipline in a voluntaristic context entails, influencing the lack of mobilisation of resources.

**Figure 1:**
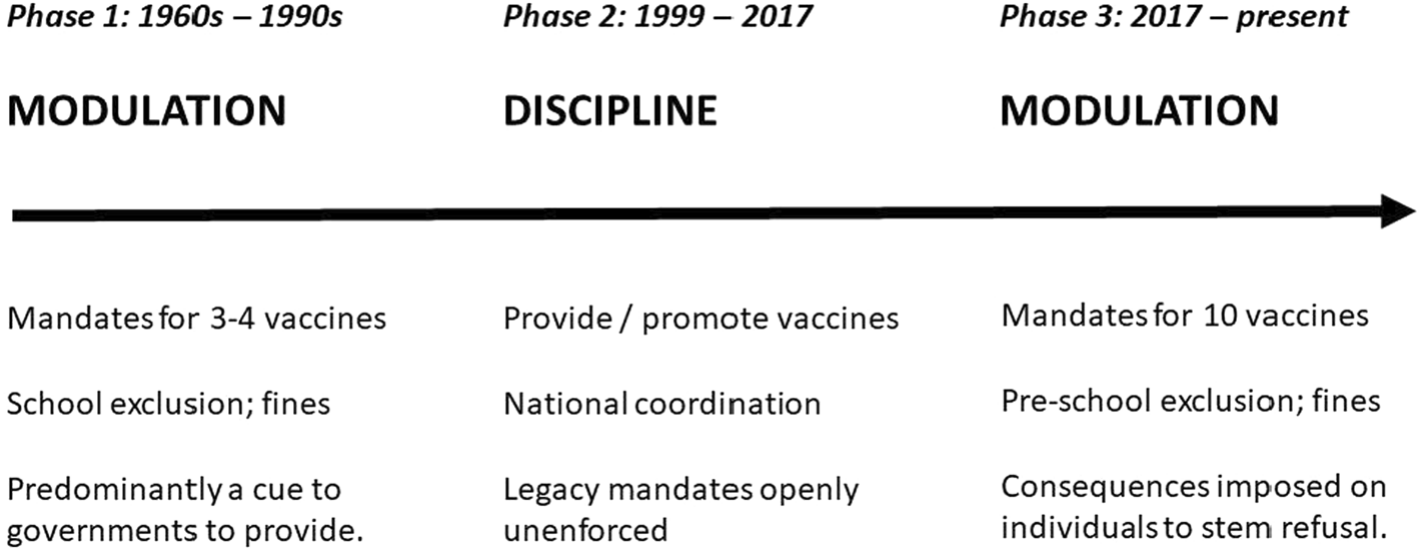
Phases of vaccination governance in Italy, 1960s–present day

Our case can help theorise compliance regime changes as the end points of inaction. Failing to employ disciplinary mechanisms adequately meant that Italian officials had to resort (back) to mandates. Legacy mandates had been ‘checkpoint’ governance, ensuring that the government was vaccinating the population. The subsequent regime expected citizens to comply with childhood vaccination because it was the ‘right’ thing to do. The government then retreated to extracting compliance by controlling access to institutions. Italy thus moved from modulation (legacy mandates) to a failed attempt at discipline (voluntarism) and then back to modulation (new mandates). These phases are illustrated in Figure 1.

Deleuzian scholars insist that modulation has not simply replaced discipline, which still operates as a method of governance. However, modulation has come to the fore in the digital age; older forms of political authority are losing their normative force as they lose control over disciplinary enclosures (Savat, 2013). The profusion of social media, as one example of this change, has meant that governments who do not ‘do communication well’ face a loss of capacity to curate the information environment in ways that are favourable for generating compliance (Marland et al., 2016), as our case study reinforces. Governments lose control over public information, as well as the identity-forming contexts through which prosocial norms can be instilled. Interviewees indicated that the UK had built a public health ‘brand’ strong enough to maintain this sort of disciplinary capacity. However, this capacity can get lost, forgotten or de-prioritised, as our case shows.

## Conclusions and recommendations

Authorities’ failure to stem Italy’s vaccination compliance problem is attributable to epistemological and organisational constraints, which made it unfeasible to assert ‘discipline’ in the face of anti-vax messaging and declining public trust. The latter were partly the result of particular circumstances in Italy, but also partly attributable to a broader decline of the authority of the state in the public sphere (Harper, 2017; Marland et al., 2016). The erosion of a unified political public sphere, combined with the erosion of government funding, credibility and planning capacity (brought on by financial and institutional precarity) directly undermined the state’s ability to maintain a ‘disciplinary enclosure’ in which it could reinforce pro-vaccination norms. This was exacerbated by under-utilisation of, or (more broadly) ineptitude regarding persuasive communications. While acute in Italy, such disciplinary breakdown is likely to be increasingly pervasive in all late capitalist democracies.

Given that Italy now enjoys higher vaccination rates, one could conclude that states facing crises in vaccination compliance can solve the problem with mandates, and extrapolate this out to other problematic areas. However, developing discipline remains manifestly valuable, especially for COVID-19 vaccination. Governments globally need to be well underway with reflexive and population-engaging strategies to vaccinate adults for whom there may never have been mandates. They will need to self-consciously engage with the disciplinary apparatus of schools, public health institutions and multi-level governance organisations, activating the “chorus” that our informant so saliently invoked. They cannot afford to misunderstand their populations, nor their information sources, nor to blame them for their distrust in institutions and expertise. And if Italy’s resort to mandates for childhood vaccines was a source of guilt, a COVID-19 mandate could be diabolical. Compulsion without significant persuasive appeals could risk people opting out altogether, as our informants identified with Italian parents purchasing non-compliance through fines. A communications-based approach that achieves sufficient compliance through discipline—the UK was the exemplar for our interviewees—adds credibility to the state’s claim to legitimacy, but requires significant resources. Elected governments need to urgently recognise the importance of investing in confidence for COVID-19 and childhood vaccines.

If it becomes too late to build compliance through voluntarism, serious consideration still needs to go into (re)building social norms. While the Italian government “telling people what to do” appears to be succeeding, mandatory vaccination makes official communications even more important. When governments compel recalcitrant individuals to vaccinate and impose consequences on holdouts, they are beholden to legitimise this decision. An optimal restrictive mandatory policy should feel coercive to the smallest possible group of people; this is only achievable by continuously confronting and delegitimising anti-vaccination sentiment. This also satisfies the imperative of addressing different motivations for compliance that exist among populations (Winter & May, 2001). Italy’s investment in vaccination promotion in schools and its ‘astronaut and sportsperson’ campaign (see p.16) represent positive steps in this direction. However, heightened compliance with childhood vaccination does not translate into confidence about COVID-19 vaccination, which 35% of Italians recently indicated they would refuse due to concerns about safety and efficacy (Ipsos, 2020).

Finally, social discipline regarding the importance of vaccination can also ‘future-proof’ compliance, with the ‘broken instruments’ scenario a lesson for when mandates are mothballed without sufficient replacement. Italy has already experienced one abortive attempt at rescinding the new mandates. Our informants regarded this as a disastrous idea given the perilous situation with measles at the time, but it demonstrates that constituencies can attack mandates for political reasons even when public health experts are tackling a crisis. Given such volatility, creating a shared social norm about the value of vaccination helps protect compliance into an increasingly uncertain future. The need for this is amplified exponentially for COVID-19 vaccination, which requires enormous investment accompanying the turmoil and suffering caused by the disease. Italy has already faced concrete challenges to vaccine acceptance for the AstraZeneca vaccine following a pause in the rollout and a pivot to recommending only for those over 60 due to a rare clotting disorder following vaccination (Guenot, 2021). Weakening disciplinary governance is likely to make modulatory control appealing to ensure COVID-19 vaccine uptake. However, all states must embrace and resource a disciplined approach to promoting and supporting vaccine compliance in the first instance and sustain it regardless of whether mandates prove tempting or necessary.

## Data Availability

The dataset generated during and analysed during the current study are not publicly available due to participant confidentiality and ethical requirements covering qualitative key informant interviews. Further information on data and analysis can be requested from the corresponding author.

## Footnotes

1 Deleuze certainly did not intend his work on control to provide a template for policymakers seeking to extract compliance from populations –he is inherently critical of such control. Nevertheless, modulation and discipline provide a useful analytical framework through which to assess and critique the operation and implications of different types of control.
